# Reduction Mammoplasty Operative Techniques for Improved Outcomes in the Treatment of Gigantomastia

**Published:** 2013-10-18

**Authors:** Brent R. DeGeorge, David L. Colen, Alexander F. Mericli, David B. Drake

**Affiliations:** Department of Plastic and Reconstructive Surgery, University of Virginia Health System, Charlottesville, Va

**Keywords:** breast reconstruction, gigantomastia, macromastia, operative techniques, reduction mammoplasty

## Abstract

**Objective:** Gigantomastia, or excessive breast hypertrophy, which is broadly defined as macromastia requiring a surgical reduction of more than 1500 g of breast tissue per breast, poses a unique problem to the reconstructive surgeon. Various procedures have been described for reduction mammoplasty with specific skin incisions, patterns of breast parenchymal resection, and blood supply to the nipple-areolar complex; however, not all of these techniques can be directly applied in the setting of gigantomastia. We outline a simplified method for preoperative evaluation and operative technique, which has been optimized for the management of gigantomastia. **Methods:** A retrospective chart review of patients who have undergone reduction mammoplasty from 2006 to 2011 by a single surgeon at the University of Virginia was performed. Patients were subdivided based on weight of breast tissue resection into 2 groups: macromastia (<1500 g resection per breast) and gigantomastia (>1500 g resection per breast). Endpoints including patient demographics, operative techniques, and complication rates were recorded. **Results:** The mean resection weights in the macromastia and gigantomastia groups, respectively, were 681 g ± 283 g and 2554 g ± 421 g. There were no differences in major complications between the 2 groups. The rate of free nipple graft utilization was not significantly different between the 2 groups. **Conclusions:** Our surgical approach to gigantomastia has advantages when applied to extremely large-volume breast reduction and provides both esthetic and reproducible results. The preoperative assessment and operative techniques described herein have been adapted to the management of gigantomastia to reduce the rates of surgical complications.

Excessive breast hypertrophy or macromastia is associated with severe pain in the back, neck, and shoulders, as well as intertrigo; problems with body image perception; and impairments with quality of life and physical functioning.^1^ The management of macromastia consists of both conservative measures and operative intervention.^2^ Conservative measures include wearing a properly fitted brassiere, physical therapy, and weight reduction.^3^ These measures have failed to demonstrate a significant improvement in relief of pain, quality of life, or patient satisfaction indices.^4^^,^^5^ Reduction mammoplasty, or surgical resection of breast parenchyma, is one of the most commonly performed plastic surgery procedures performed in the United States, with more than 130,000 cases reported in 2010.^5^^-^7 Reduction mammoplasty has been shown to improve or eliminate pain, restores physical activity and quality of life, and has one of the highest patient satisfaction rates of any procedure performed by plastic surgeons.^8^^,^ 9

Gigantomastia, or excessive breast hypertrophy, which is broadly defined as macromastia requiring reduction of more than 1500 g of breast tissue per breast, poses a unique problem to the reconstructive surgeon.^10^^,^^11^ Various procedures have been described for reduction mammoplasty with specific skin incisions, patterns of breast parenchymal resection, and retained blood supply to the remaining breast tissue and areolar complex; however, not all of these techniques can be applied successfully in the setting of gigantomastia.^4^^,^^12^^-^16

To date, only small case reports and series are available which specifically address the surgical management of this specific group. We seek to elucidate the patient-specific, breast-specific, and procedure-specific elements of reduction mammoplasty associated with improved outcomes in the setting of gigantomastia. A retrospective chart review of patients who have undergone reduction mammoplasty from 2006 to 2011 with particular attention to the cohort of patients who have undergone resection of more than 1500 g of breast tissue per breast by a single surgeon at the University of Virginia was performed. We outline a simplified approach for patient preoperative evaluation and marking as well as surgical resection and breast reconstruction, which have proven advantageous when applied to extremely large-volume breast reduction and provided both esthetic and reproducible results. These operative techniques have been adapted to the management of gigantomastia to reduce the rates of surgical complications, including minor complications such as asymmetry, hematoma, seroma, focal superficial skin necrosis, nipple-areolar complex hypopigmentation, and major complications such as reoperation and nipple-areolar complex necrosis. We intend to use the data gathered from this study to generate an algorithm for systematic patient evaluation and operative planning for the management of gigantomastia.

## METHODS

The methods described herein were approved by the University of Virginia institutional review board. All female patients aged 18 years or older who underwent bilateral reduction mammoplasty between July 2006 and June 2011 were reviewed. The senior author (D.B.D.) performed all of the procedures assisted by plastic surgery residents. For the purposes of this study, gigantomastia is defined as more than 1500 g of breast tissue resected per breast, which is consistent with other reports.^10^ Patients were subdivided on the basis of weight of breast tissue resection into 2 groups: macromastia (<1500 g resection per breast) and gigantomastia (>1500 g resection per breast). We included patients who had a 1500 g of breast tissue resected from at least 1 breast for data analysis. Patient demographic factors, comorbidities, preoperative body mass index, and weight of the resected breast tissue were recorded. Sixty-seven patients underwent bilateral reduction mammoplasty during the study time period, and 11 patients were identified with gigantomastia from review of operative and pathology reports. Review of medical records from initial patient clinical encounters revealed that all patients reported some degree of pain in back, neck, or shoulder as well as intertrigo of the inframammary fold; bilateral brassiere strap grooving; and dissatisfaction with breast esthetic appearance, contour, and size. Demographic data are detailed in Table 1. Women with gigantomastia were significantly more obese with an average body mass index of 45.1 ± 2.9 versus 32.8 ± 0.9 (*P* = .0006). There were other minor yet significant differences in between groups in that women with gigantomastia were more frequently hypertensive and tended to be younger at the time of mammoplasty.

## RESULTS

The weights of resected specimens ranged from 300 g to 1350 g per breast in the nongigantomastia group with a mean resection weight and standard deviation of 681 g ± 283 g, and in the gigantomastia group the weights of resected specimens range from 1500 g to 7050 g per breast with a mean resection weight and standard deviation of 2554 g +/−421 g, which was significantly different between groups (*P* < .001) (see Table 1). Overall, there were no differences in major complications between reduction mammoplasty procedures in the gigantomastia versus macromastia groups (see Table 2). Minor complications were more frequent in the gigantomastia group, and the majority of these involved the nipple-areolar complex appearance; however, the rate of total nipple-areolar complex loss was not significantly different between the 2 groups. The method most commonly used for reduction mammoplasty avoids the need for free nipple graft in both groups, and the rate of free nipple graft utilization was not significantly different between the 2 groups (*P* = .11); however, when this technique was used, it accounted for the increased incidence of nipple-areolar complex hypopigmentation and partial necrosis.

## DISCUSSION

**Figure 1:**
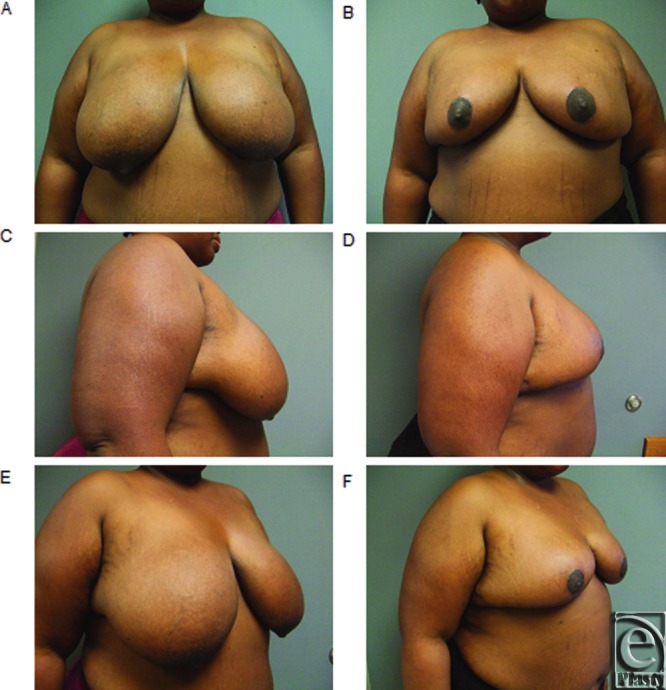
(*a*-*f*) Representative preoperative and postoperative breast photograph series in patient with gigantomastia demonstrating “no vertical scar” reduction mammoplasty.

**Figure 2:**
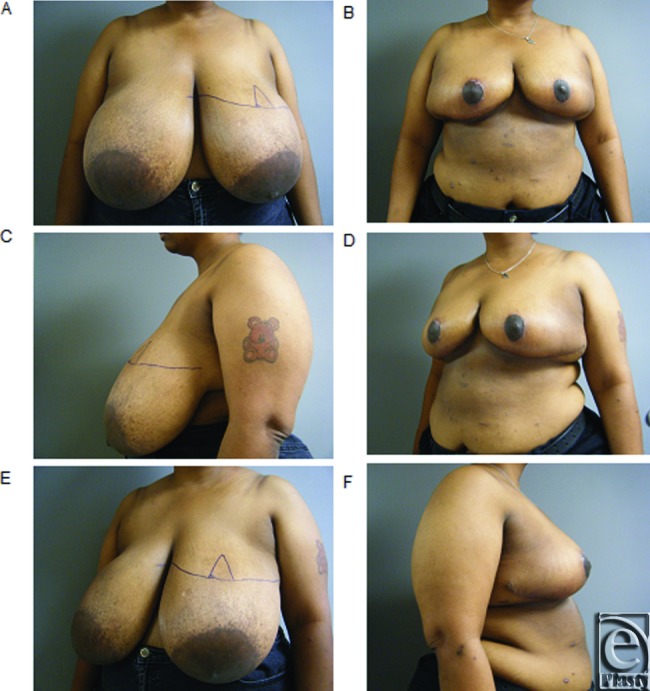
(*a*-*f*) Representative preoperative and postoperative breast photograph series in patient with gigantomastia demonstrating “no vertical scar” reduction mammoplasty.

The individual techniques used in this article for the management of gigantomastia have been described singly elsewhere; however, the aggregate of techniques described herein has been optimized by the senior author to provide a safe and effective means of large-volume breast reduction while preserving breast contour and projection. The reductionist breast surgeon should be familiar with a number of approaches and techniques and not constrained by one technical approach applied to each patient. In this series, a standard approach was utilized, but easily altered or modified depending on the desires of the patient as well as breast size and shape. Most commonly, an inferior pedicle technique utilizing a standard wise pattern skin incision was used. Secondarily, a “no vertical scar” technique as described by Passot and refined by Lalonde was next most commonly used (Figs 1 and 2).^17^^,^^18^ The advantages of the “no vertical scar” approach are both esthetic and functional. This technique allows for placement of scars at the breast esthetic subunit boundaries of the areola and the inframammary fold and avoids the necessity of the vertical T limb at closure, which in this patient population has increased risk of unsightly scarring, skin necrosis, and wound breakdown.^19^ The downside of this technique is that it tends to make a more rounded breast mound without as much central projection. However, this technique was performed on most of the larger breasts.

The utility of the inferior pedicle technique in this patient population is 2-fold: it can be used on any length breast and is not constrained by measurements. We feel that the paramount determinant of nipple viability is the preservation of the inferior perforators from the chest wall, and not the length of the dermal pedicle. The measurement of sternal notch to areola or inframammary fold to nipple distance was not used to determine the approach to the patient. It is important to note that in these large breasts, a dermal pedicle that is planned too wide in an attempt to preserve nipple-areolar complex viability can actually be detrimental to blood flow, both to the pedicle and the overlying skin envelope. Closure of the skin envelope can result in excessive tension on an overly bulky dermal pedicle, and in this instance, additional central pedicle may need to be resected to allow for closure without excessive tension.

The technique of breast reduction most commonly used in this series was an inferior-based pedicle technique. However, each breast configuration is different and this technique is a free-form technique that allows the surgeon to modify, shape, and adjust each breast to optimize shape and contour. To achieve successful results with this free-form approach, we have outlined the following general principles for preoperative planning, intraoperative techniques for breast parenchyma resection and shaping, and closure.

### Preoperative markings

Typical breast marking always occurs with the patient sitting upright and straight (Fig 2). The suprasternal notch is marked, followed by lateral marks placed on the clavicle at the junction of the medial and middle third. This typically measures between 6 and 7 cm depending on the patient's body habitus. The apex point is then chosen at the level of the inframammary fold. In patients with true gigantomastia, this commonly measures at 23 cm or greater. It is important to realize that the inframammary fold may be displaced inferiorly by the weight of the gland and a standard number should not be used. This apex point is placed at the central meridian of the breast and not necessarily on the center of the gland. The central meridian of the breast is usually located within 12 cm of the midline. Commonly in these large-breasted patients, the weight of the breast parenchyma has caused the breast to prolapse laterally, and the majority of the gland is not under the central meridian of the breast. It is important while marking to center the breast on the chest wall by moving it medially. Following selection of the apex point, 7-cm limbs are then marked to create a triangle with a base of 5 cm. Depending on the patient and amount of gland resected, the surgeon has the option of either removing the triangle or keeping it for a “no vertical scar” technique. The authors have found that the “no vertical scar” technique has a lower complication rate, primarily due to avoidance of the excessive tension and decreased blood flow on the vertical limbs and the “T” point. However, this usually leads to less central projection from the skin envelope shaping and some lack of narrowing the base of the breast. If the patients are counseled preoperatively about the risks, benefits, and alternatives of an “inverted T” versus a “no vertical scar,” including the differences in expected breast shape, most are accepting of this issue. In our hands, the wise pattern gives a more youthful appearance while the “no vertical scar” a more mature breast shape. Furthermore, for the obese patient, it is helpful during preoperative counseling to draw attention to the extra skin folds of skin and fat that extend laterally under the arms and many times well posteriorly. This tissue is not routinely part of the reduction in these morbidly obese patients, and with the repositioning of the new breast the prominence of these folds may be accentuated and become more noticeable to the patient. Identifying this issue and discussing it with the patient preoperatively is important for patient satisfaction.

### Flap elevation and parenchymal resection

The superior skin flap ultimately will be redraped over the dermal pedicle and new breast mound. To achieve a uniform contour, it is important to thin out the flap centrally to accept the bulk of the pedicle that will be placed under this tissue. Typically, our superior flap is thinned to between 1.5 and 2 cm in the area overlying the dermal pedicle. Further dissection in this region may compromise blood supply to the skin flap. Dissection proceeds superiorly to the level of the subclavicular pectoralis major muscle, which is important to allow for tension-free re-draping of the skin flap over the new breast mound. Avoidance of medial perforator resection is critical to the success of this maneuver so as not to de-vascularize or render ischemic this large flap.

**Figure 3:**
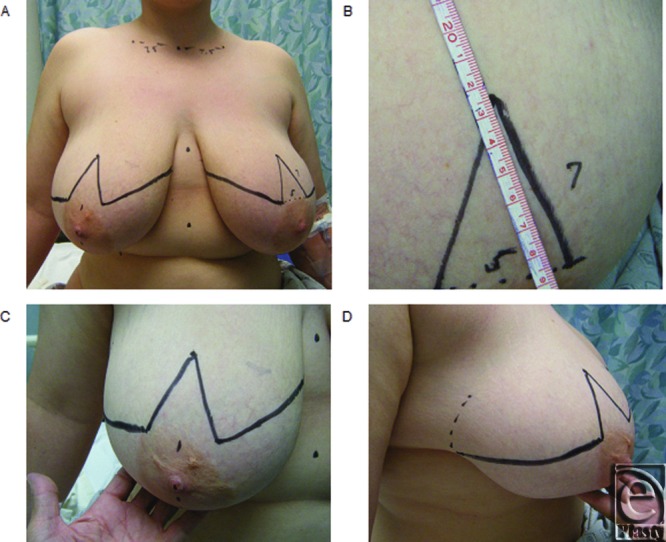
Representative intraoperative reduction mammoplasty photographs. (*a*) Markings for skin and breast parenchymal resection. (*b*) Elevation of the superior flap. Note that the area between the surgeon's left hand must be thinned to accommodate the dermal pedicle. (*c*) Elevation of the pedicle with preservation of chest wall perforators. (*d*) Placement of shaping sutures from the pedicle to the chest wall to facilitate central projection.

Resection of the gland occurs in a stepwise fashion with the lateral segment removed first, followed by the medial segment. Often the lateral segment is large in these patients and the medial segment is small. The segments are resected in a triangular fashion with the segments removed from the superior flap straight down to the chest wall and medially with pedicle supported so as not to undermine this central mound. The base width of the dermal pedicle is chosen at 6 to 8 cm no matter the size of the resection and centered on the breast meridian. The vascularity of the nipple-areolar complex is determined by the vascular perforators feeding the pedicle from the chest wall, and not predominately by the dermal skin paddle. Over-resection of the pedicle base can lead to nipple-areolar ischemia or necrosis, whereas under-resection may cause nipple-areolar complex ischemia due to closure with a tight skin envelope (Fig 3).

### Optimization of breast projection and contour

Previously described amputation techniques tend to leave a flat central breast in this patient population, as most of the gland has fallen beneath the inframammary fold. Rebuilding the central mound and repositioning it on the chest wall is a necessity in these patients. Using internal sutures to shape the breast mound has become an essential component to the surgical technique in these patients. The principal benefits of shaping sutures are to facilitate central projection and to prevent the bulk of the long pedicle from displacing laterally. To this end, sutures are placed in a manner to tack the breast parenchyma medially and may occasionally be placed to secure the pedicle itself medially to the chest wall to achieve maximal central projection, and to keep the breast mound central to the axis of the breast (Fig 3). Care must be taken not to restrain the nipple-areolar complex from assuming the new chosen position on the chest wall. Most shaping sutures are done near the base and are loosely tied. For breast shaping, we typically use interrupted 2-0 polydioxanone sutures for retention of tensile strength. Assessment of the final shape and symmetry should always be judged in the upright position and revised until approximate symmetry and shape is achieved.

### Placement of the nipple-areolar complex and closure

**Figure 4:**
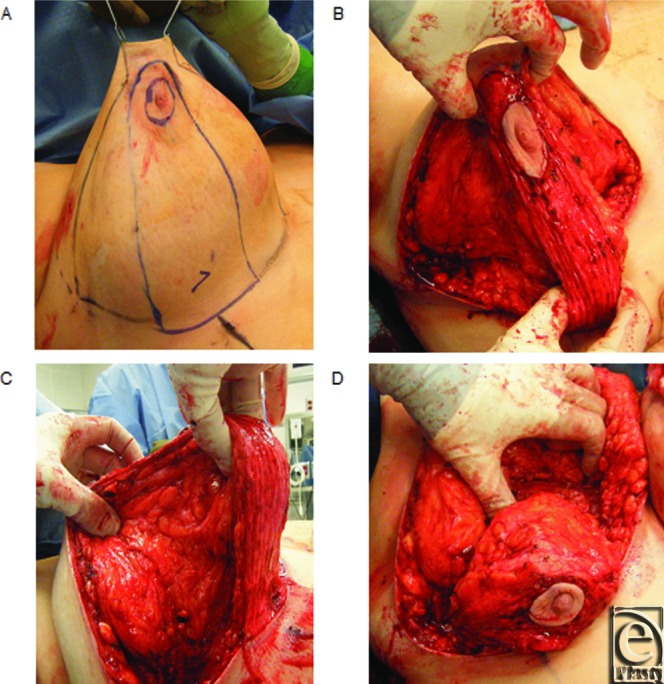
Representative preoperative reduction mammoplasty markings. (*a*) Overview of markings. (*b*) Marking of the apex point. (*c* and *d*) Marking of the apex point and resection triangles. Note the surgeon's hand is used to correct the lateral displacement of the gland prior to marking.

Using the triangle method of marking described earlier has the advantage of placing the nipple in the most esthetically pleasing location, and not being committed to a particular nipple location as in the case when the McKissock keyhole pattern is used.^20^ On occasion the most appropriate nipple location does not match the vertical limb of the inverted T and the flexibility to position the nipple-areolar complex in the most pleasing location on the central mound is an advantage of this approach (Fig 4. This position is chosen at 4.5 to 5 cm from the inframammary fold to the bottom of the areola to allow for some bottoming out in these patients. Closure of the nipple-areolar complex is accomplished by choosing the appropriate position and size. Nipple diameter is initially selected at 42 cm and inset at 38 cm to 40 cm depending on ultimate breast size and patient preference. This circle is then de-epithelialized and the dermis is then incised radially from the 9 to 3 o’clock position and from the 6 to 12 o’clock position. Occasionally, subdermal fat will be resected when excessive to facilitate delivery of the nipple-areolar complex and closure. The inferior pedicle is delivered without tension and sutured in a sequential fashion at the cardinal directions, and then these are bisected with 4-0 polyglactin 910 interrupted sutures, followed by a running suture of 5-0 polyglactin 910 and supported with cyanoacrylate skin adhesive to avoid any suture marks. The inframammary fold is then approximated in similar fashion with 3-0 polyglactin 910 interrupted sutures followed by a 4-0 polyglactin 910 running subcuticular closure.

### Pearls and pitfalls

The 4 most common pitfalls that we have identified with this approach are as follows: (1) leaving the central pedicle too wide, large, and bulky to facilitate a tension-free closure; (2) failure to place glandular shaping sutures to optimize central breast projection and prevent lateral parenchymal displacement; (3) tying shaping sutures excessively tight—these sutures are designed to support the breast parenchyma in place, and excessive tension will result in strangulation of the parenchymal tissue and not allow for tension-free delivery of the nipple-areolar complex; and (4) failure to tailor the superior skin flap to accommodate the shape of central pedicle.

Preoperative counseling is of paramount importance in the case of gigantomastia. It is important to convey the notion that breasts are similar, but not identical. Although symmetry is important, exact symmetry is impossible to achieve for a variety of reasons. It is important to note preoperative breast asymmetries, chest wall conditions, such as pectus excavatum or carinatum, and scoliosis, as these can become more apparent postoperatively, and these variations in body habitus have the capacity to negatively affect apparent breast position. In small breasts, breast asymmetry may not be as noticeable. However, when examined closely in patients with gigantomastia, the breast differences are magnified. Many patients have not inspected their breasts to the degree that they have actually noticed these preoperative differences in breast volume and shape. Identifying and communicating these asymmetries preoperatively to the patient is an important component of informed consent. Some adjustment for asymmetry can be made by careful parenchymal excision; however, in the majority of cases some degree of asymmetry will remain.

### Summary

We present one surgeons’ approach to breast reduction in the setting of gigantomastia detailing some common pitfalls and issues that should be addressed. There are many described methods for the surgical management of these patients with most leading to acceptable patient satisfaction. In our hands, this simplified approach can produce a safe, predictable outcome with complication profile similar to nongigantomastia reductions.

## Figures and Tables

**Table 1:** Demographic factors, comorbidities, and resection volumes in gigantomastia and macromastia patient populations prior to reduction mammoplasty

Demographic factor	Gigantomastia	Macromastia	*P*
Age, y	42.6 ± 11.6	37.2 ± 13.2	.218
BMI, kg/m^2^	45.1 ± 9.2	32.8 ± 6.4	.002
Comorbidity			
HTN, %	6 (40)	9 (60)	.013
DM, %	3 (75)	1 (25)	.013
COPD, %	0	0	NA
CAD, %	0	0	NA
OSA, %	1 (100)	0	.12
Smoker, %	2 (18)	9 (82)	1
Prior reduction, %	0 (0)	3 (100)	1
Prior breast surgery, %	1 (13)	7 (87)	1
Resection volume			
Mean, g	2554 ± 421	681 ± 283	<.001
Range, g	1500-7050	300-1350	<.001

BMI indicates body mass index; CAD, coronary artery disease; COPD, chronic obstructive pulmonary disease; DM, diabetes; HTN, hypertension; OSA, obstructive sleep apnea.

**Table 2:** Complication rates in gigantomastia and macromastia patient populations following reduction mammoplasty

Complication	Gigantomastia (%)	Macromastia (%)	*P*	Odds ratio gigantomastia	Odds ratio macromastia	95% CI
Superficial skin necrosis	0 (0)	4 (7)	1	NA	0.82	.73-.92
Cellulitis	2 (18)	7 (13)	.64	1.1	0.72	.26-8.4
Hematoma minor	0	0	NA	NA	NA	NA
Seroma minor	1 (9)	2 (4)	.43	1.3	0.42	.56-2.8
Wound dehiscence/breakdown	2 (18)	4 (7)	.25	1.3	0.44	.72-2.2
Nipple hypopigmentation	3 (27)	1 (2)	.012	3.5	0.17	.64-19
Partial nipple necrosis	2 (18)	1 (2)	.07	2.5	0.22	.52-12.7
Any minor	8 (73)	16 (29)	.013	1.3	0.21	1-1.8
Reoperation	1 (9)	4 (7)	1	1	0.8	.6-1.6
Total loss of nipple-areolar complex	0	0	NA	NA	NA	NA
Admission for IV antibiotics	0	0	NA	NA	NA	NA
Hematoma requiring surgery	0	0	NA	NA	NA	NA
Free nipple graft loss	0	0	NA	NA	NA	NA
Any major	1 (9)	4 (7)	1	1	0.8	.6-1.6
